# Jabba: hybrid error correction for long sequencing reads

**DOI:** 10.1186/s13015-016-0075-7

**Published:** 2016-05-03

**Authors:** Giles Miclotte, Mahdi Heydari, Piet Demeester, Stephane Rombauts, Yves Van de Peer, Pieter Audenaert, Jan Fostier

**Affiliations:** Department of Information Technology, Ghent University - iMinds, Ghent, Belgium; Department of Plant Systems Biology, VIB, Ghent, Belgium; Department of Biotechnology and Bioinformatics, Ghent University, Ghent, Belgium; Bioinformatics Institute Ghent, Ghent, Belgium; Department of Genetics, Genome Research Institute, University of Pretoria, Pretoria, South Africa

**Keywords:** Sequence analysis, Error correction, de Bruijn graph, Maximal exact matches

## Abstract

**Background:**

Third generation sequencing platforms produce longer reads with higher error rates than second generation technologies. While the improved read length can provide useful information for downstream analysis, underlying algorithms are challenged by the high error rate. Error correction methods in which accurate short reads are used to correct noisy long reads appear to be attractive to generate high-quality long reads. Methods that align short reads to long reads do not optimally use the information contained in the second generation data, and suffer from large runtimes. Recently, a new hybrid error correcting method has been proposed, where the second generation data is first assembled into a de Bruijn graph, on which the long reads are then aligned.

**Results:**

In this context we present Jabba, a hybrid method to correct long third generation reads by mapping them on a corrected de Bruijn graph that was constructed from second generation data. Unique to our method is the use of a pseudo alignment approach with a seed-and-extend methodology, using maximal exact matches (MEMs) as seeds. In addition to benchmark results, certain theoretical results concerning the possibilities and limitations of the use of MEMs in the context of third generation reads are presented.

**Conclusion:**

Jabba produces highly reliable corrected reads: almost all corrected reads align to the reference, and these alignments have a very high identity. Many of the aligned reads are error-free. Additionally, Jabba corrects reads using a very low amount of CPU time. From this we conclude that pseudo alignment with MEMs is a fast and reliable method to map long highly erroneous sequences on a de Bruijn graph.

## Introduction

### Background

The accurate determination of the DNA sequence of an organism, i.e., establishing the precise order of the nucleotides A, C, G and T in a DNA molecule, is a fundamental and challenging problem in biology. Essentially this process consists of two steps: (1) sequencing the DNA by means of a chemical process, resulting in a large number of reads and (2) genome assembly, where the reads are processed to reconstruct the complete DNA sequence. Every sequencing technology results in reads that contain errors, with error profiles varying greatly between platforms. There is a clear distinction between *second generation* reads and *third generation* reads, where the latter are characterized by vastly improved read lengths albeit with much higher error rates.

For second generation sequencing we mainly consider the Illumina platform. The different Illumina technologies produce many short (100–300 nucleotides) reads with a high accuracy ($<$2 % errors, mainly substitutions) with high throughput and at a low financial cost. New algorithms, based on de Bruijn graphs, were specifically developed to efficiently deal with the assembly of huge amounts of second generation sequencing data. Overlap between short reads is then established in linear time between reads that share a *k*-mer, i.e., a substring of length *k*. Repeat resolution in the de Bruijn graphs is however severely hindered by the very short read length of the second generation data.

**Table 1:** The data sets and reference genomes

	ID	Number of reads	Number of bases (Mbp)	Maximal read length	N50	Estimated coverage
*Escherichia coli*						
Reference	NC_000913^a^					
Short reads	ART	28.4 M	2840	100	100	600×
Long reads	SRR1284073^b^	163 K	649	49,424	13,578	135×
*Aeromonas hydrophila*						
Reference	NC_008570^a^					
Short reads	ART	4.74 M	474	100	100	100×
Long reads	*pbsim*	515	4.74	24,430	10,421	1×
*Saccharomyces cerevisiae*						
Reference	NC_001133^a^					
Short reads	ART	9.72 M	2430	250	250	200×
Long reads	SRR1284074^b^	1.96 M	5580	37,008	3973	453×
	SRR1284662^b^					
*Ostreococcus tauri*						
Reference	NC_014426^a^					
Short reads	[30]	9.72 M	1778	76	76	135×
Long reads	[30]	225 K	1135	22,892	7322	86×
*Arabidopsis thaliana*						
Reference	NC_003070^a^					
Short reads	ART	23.9 M	5975	250	250	49×
Long reads	SRR1284093^b^	327 K	1439	86,350	14,256	12×
	SRR1284094^b^					
*Drosophila melanogaster*						
Reference	Release 5^c^					
Short reads	ART	24.1 M	6025	250	250	49×
Long reads	SRR1204085^b^	327 K	686	55,988	12,478	6×
	SRR1204086^b^					

^a^Reference genome available at http://www.ncbi.nlm.nih.gov/nuccore

^b^Reads available at http://www.ncbi.nlm.nih.gov/sra

^c^Reference genome available at http://www.fruitfly.org/sequence/release5genomic.shtml

Recently, third generation sequencing technologies (Pacific Biosciences, 2013; Oxford Nano Technologies, 2014) began to emerge. Pacific Biosciences SMRT sequencing results in much longer reads (avg. >5000 nucleotides), albeit with significantly higher error rates (up to 15%, mostly insertions and deletions and to a lesser extent substitutions). Despite this high error rate, a very high consensus accuracy may be achieved because the errors are uniformly distributed over the read. If the coverage is sufficiently high and overlap between the reads is correctly established, this uniform distribution of errors allows for very accurate consensus calling. Computing these overlaps can not be efficiently achieved by means of a de Bruijn graph, because the high error rate leads to an overabundance of incorrect *k*-mers. Therefore, other efficient methods have been developed to compute pairwise alignments between third generation reads [1, 2].

### Error correction

The processing of sequencing reads usually involves mapping them to other sequences, either by aligning the reads to each other to establish potential overlap, or by mapping them to a reference genome. Errors in the reads introduce noise to these alignments, leading to weaker alignments than the corresponding error-free reads would have. Lower rated alignments may then be discarded for further analysis, potentially discarding crucial information. This can be especially problematic when dealing with low quality reads in a region with low coverage. To deal with this sequencing noise, error correction methods can be applied. By correcting the errors in the reads, the optimal alignments can be more accurately identified and more appropriately rated, leading to better downstream analysis, as shown in e.g. [3] for de novo assembly.

**Table 2:** Results for LoRDEC, proovread and Jabba

	Gain (%)	Accuracy (%)	Error-free (%)	Aligned (%)	Throughput (%)	N50 (bp)
*E. coli* - simulated short and real long reads - 4.7 Mbp						
Uncorrected reads		85.16	0	59.16		13,578
LoRDEC_n_	96.46	99.47	13.74	82.16	62.30	4661
LoRDEC	98.83	99.82	79.31	88.95	63.70	7618
proovread	99.64	99.94	89.64	99.57	58.65	5706
Jabba	99.70	99.95	95.70	99.23	57.04	12,760
*A. hydrophila*—simulated short and simulated long reads: 4.8 Mbp						
Uncorrected reads		86.84	0	100		10,421
LoRDEC_n_	99.21	99.89	25.29	96.72	94.79	7625
LoRDEC	99.93	99.99	86.74	99.76	95.35	9695
proovread	99.99	99.99	96.53	99.99	95.40	9803
Jabba	99.74	99.96	97.66	99.98	98.04	10,215
*S. cerevisiae* - simulated short and real long reads: 12.3 Mbp						
Uncorrected reads		83.21	1.50	27.99		3969
LoRDEC_n_	91.17	98.51	44.02	77.77	21.72	2869
LoRDEC	92.08	98.67	60.82	83.12	30.43	3802
proovread	–	–	–	–	–	–
Jabba	99.87	99.97	98.35	99.93	27.67	8373
*O. tauri* - real short and real long reads: 13.2 Mbp						
Uncorrected reads		83.83	0.05	23.10		7322
LoRDEC_n_	91.04	98.55	63.60	85.05	31.43	985
LoRDEC	91.51	98.62	66.76	85.42	31.54	1043
proovread	98.11	99.69	80.28	90.55	26.31	1501
Jabba	99.06	99.84	83.33	93.31	13.81	4183
*A. thaliana* - simulated short and real long reads: 121 Mbp						
Uncorrected reads		83.32	8.00	47.82		14,256
LoRDEC	90.43	98.40	59.35	50.69	46.09	904
proovread	91.11	98.51	69.71	96.66	42.08	7788
Jabba	99.47	99.91	96.67	99.85	39.87	12,647
*D. melanogaster*—simulated short and real long reads: 122 Mbp						
Uncorrected reads		85.70	22.97	41.72		12,478
LoRDEC	89.18	98.45	54.29	49.24	44.78	1119
proovread	97.07	99.58	67.72	98.36	43.49	11,476
Jabba	99.51	99.93	96.24	99.81	38.20	15,553
Jabba_p_	99.51	99.93	96.24	99.82	38.22	15,564

Results for proovread on *S. cerevisiae* have been left out because they did not compute in 3 days. The subscript *p* indicates that the tool used the reference genome instead of short reads. The subscript *n* indicates that the tool used uncorrected short reads

Algorithms to correct second generation reads have been classified [4] into three types. The *k*-mer spectrum-based methods [5, 6] rely on coverage thresholds to determine whether a *k*-mer represents part of the actual DNA sequence. The suffix tree-based methods [7, 8] generalize the *k*-spectrum methods by handling multiple *k* values at once. Finally, the multiple sequence alignment-based methods [9] correct the reads after aligning several similar reads.

To correct third generation reads, they can be aligned to each other and a consensus sequence between overlapping reads may then be computed. However, the coverage required for high accuracy consensus-based correction of third generation reads can lead to a prohibitively high financial cost for many sequencing projects. Hybrid error correction methods provide an alternative. The goal is to correct long third generation reads using the more accurate sequence information contained in second generation reads. The idea is that a (relatively cheap) second generation data set might be sufficient to correct the long reads, regardless of the coverage of third generation data. This may result in a reduced financial cost for sequencing, as low coverage third generation data might suffice. Hybrid error correction methods also appear attractive from a computational point of view as they avoid pairwise comparisons between long reads, thus circumventing the quadratic computational complexity. The first type of hybrid error correction methods LSC [10], PacBioToCA [11] and proovread [12] rely on mapping short reads to long reads, and then calling the consensus sequence from this multiple alignment. However, such methods map short reads individually and do not exploit the context in which the short read occurs. A more recent hybrid error correction method, LoRDEC, first constructs a de Bruijn graph from the short reads and then maps the long reads on this graph. The sequence implied by the path in the graph to which the long read aligns then represents the corrected read. The use of a de Bruijn graph has the advantage that overlap between short reads is established prior to mapping them to long reads. In [13], it was shown that LoRDEC achieves similar accuracy as other error correction methods, but with significantly improved runtimes. LoRDEC uses a *k*-mer index where every seed corresponds to a node in the graph.

**Table 3:** Average CPU time per read for LoRDEC, proovread and Jabba

	LoRDEC (ms)	proovread (ms)	Jabba (ms)
*E. coli*: 4.7 Mbp	111	1782	47
*A. hydrophila:* 4.8 Mbp	582	5652	11
*S. cerevisiae*: 12.3 Mbp	172	–	28
*O. tauri*: 13.2 Mbp	462	3165	9
*A. thaliana*: 121 Mbp	633	2128	100
*D. melanogaster:* 122 Mbp	289	1699	53

Results for *proovread* on *S. cerevisiae* have been left out because they did not compute in 3 days

We introduce Jabba, a hybrid error correction method for third generation reads. In Jabba, third generation reads are mapped to a de Bruijn graph [14] built from second generation reads, using a pseudo alignment approach based on a seed-and-extend methodology. The resulting paths in the graph dictate the read correction. The seeds are maximal exact matches (MEM) between an individual read and a node of the graph.

The usage of MEMs as seeds has several advantages over *k*-mers as they are used in LoRDEC. Firstly, the seeds can be longer. Even though long seeds only occur rarely, a few longer seeds can be sufficient to have a rough estimate of how the read should be aligned to the graph. Shorter seeds can then be used to further refine this. Secondly, given an enhanced suffix array [15], seeds of arbitrary lengths can be sought without the need to rebuild this index. This is not the case for a *k*-mer index (e.g. a hash table): when different values for *k* have to be used during the alignment process, different *k*-mer indexes need to be built of the graph. Finally, the use of MEMs allows for the use of arbitrary values of *k* to build the de Bruijn graph. Since the high error rates of the third generation reads are the limiting factor on the minimal seed size, this offers a clear advantage over the state of the art in hybrid error correction. This decoupling of seed size and *k*-mer size allows the use of a larger value of *k* to build the de Bruijn graph, resulting in a less complex de Bruijn graph. The *k*-mer size of the de Bruijn graph is then limited by the error rate in the second generation data. In this way, correcting the short reads before constructing the graph and using MEMs as seeds act together in allowing large *k*-values for the de Bruijn graph, effectively resolving many small repeats.

**Table 4:** Peak memory usage for LoRDEC, proovread and Jabba

	LoRDEC (MB)	proovread (MB)	Jabba (MB)
*E. coli* - 4.7 Mbp	2946	17,035	175
*A. hydrophila* - 4.8 Mbp	1205	617	103
*S. cerevisiae* - 12.3 Mbp	2693	–	401
*O. tauri* - 13.2 Mbp	2208	12,963	328
*A. thaliana* - 121 Mbp	3876	7042	5098
*D. melanogaster* - 122 Mbp	3936	6656	4099

Results for *proovread* on *S. cerevisiae* have been left out because they did not compute in 3 days

Jabba is implemented in C++ and OpenMP. The source code, installation instructions, and manual are freely available at http://bioinformatics.intec.ugent.be/jabba.

## Methods

### Overview

In this work, we further build upon the idea of using a de Bruijn graph for hybrid error correction of long reads. Specifically, the main goal is the use of Illumina data to correct Pacific Biosciences SMRT reads.

**Fig. 1:**
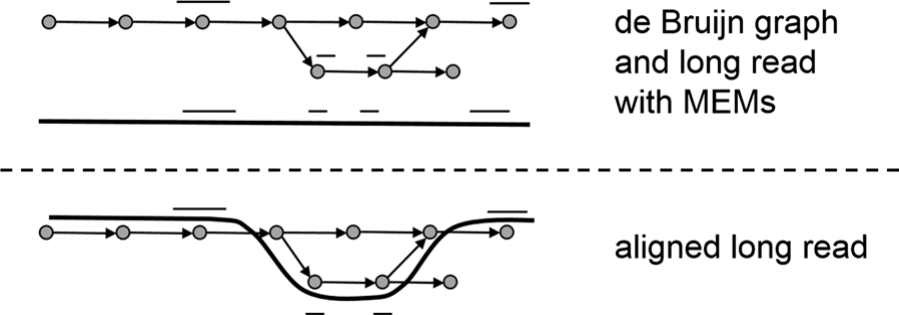
To align a read to the de Bruijn graph, a seed-and-extend algorithm is used. First MEMs are found between the read and the graph, then a path in the graph is found between these seeds, creating the final alignment

To this end, the Illumina data is corrected using existing tools (e.g. Karect [16]). From the corrected Illumina data a de Bruijn graph is constructed and this graph is then further corrected using standard procedures [17]. Subsequently, long reads are aligned along a path in the graph. This path then dictates the correction of the long reads. This procedure is summarized in Fig. 1.

Whereas LoRDEC relies on shared *k*-mers to align the long reads to a de Bruijn graph, we explore the idea of using maximal exact matches (MEMs). MEMs are exact matches between two sequences that can not be extended in either direction. This as opposed to common *k*-mers, which are exact matches of a fixed length *k*, which may or may not be maximal. Alignment methods based on maximal exact matches have been developed for read mapping [18–20]. It is shown in [18] that these methods can be more efficient than alignment techniques based on *k*-mers and Burrows–Wheeler transforms [21, 22]. From the definition of a MEM, it is clear that every MEM of size $l\ge k$ can be represented as a consecutive sequence of *k*-mers, and vice versa. However, finding large MEMs can be achieved in an efficient manner, and MEMs can compactly represent multiple *k*-mers.

**Table 5:** Peak memory usage for the index in Jabba, with different sparseness factors on *A. hydrophila*

Sparseness factor	Memory (MB)
1	103
2	62
3	48
4	41

The remainder of this section is dedicated to a more in-depth description of all steps involved.

### Assembly of the second generation data

Before the main error correction procedure can start, the second generation data is assembled in a de Bruijn graph. In the Jabba workflow this preprocessing step has two phases, first the reads are corrected, then a de Bruijn graph is constructed from these corrected reads.

#### Two phase preprocessing

In the preprocessing phase for Jabba, the second generation reads are processed twice. First a relatively small *k*-mer size (e.g. $k=13$) is used to correct the reads, using Karect [16]. The resulting reads have a very high per base quality and these are then used to build a de Bruijn graph with a relatively high *k*-mer size (e.g. $k=75$). On this graph further corrections can then be performed, as described below. This approach has two main advantages:

1. The per base accuracy is very high, which is crucial since the long reads are corrected based on the node content of the de Bruijn graph.
2. Repeats smaller than the *k*-mer size are resolved in the de Bruijn graph. For large values of *k* (e.g. $k=75$ for 100 bp reads) this greatly reduces the complexity of the graph, which facilitates the alignment of sequences to the graph.

#### Graph correction

Errors in short reads lead to erroneous paths in the de Bruijn graph. Three types of errors can be discerned based on their position in the read. An error that is located at least $k-1$ nucleotides away from both ends of the read will result in *k* erroneous *k*-mers. In turn, this leads to the formation of a ‘bubble’, i.e. a path of length *k* that runs parallel to the real path. On the other hand, errors positioned close to the ends of the read lead to the creation of less than *k* erroneous *k*-mers, thus forming ‘dead ends’ (tips) in the de Bruijn graph. Errors in the reads may also result in chimeric connections between unrelated parts of the graph. Additionally, because of coverage biases certain paths could be absent or underrepresented in the graph.

**Table 6:** Runtimes and peak memory usage for Karect, with a limit of 64 Gb memory

	CPU time (h)	Memory (GB)
*E. coli*	5.26	35.0
*A. hydrophila*	3.57	9.7
*S. cerevisiae*	1.99	60.4
*O. tauri*	1.60	37.7
*A. thaliana*	18.27	50.3
*D. melanogaster*	16.01	50.6

These errors can be corrected as described in [17]. Assuming a sufficiently low error rate and a high coverage, the correct path in a bubble will typically have a higher coverage than parallel erroneous paths, and the graph can be corrected by removing the erroneous path. Tips can be easily identified and removed, based on topology and coverage considerations. The chimeric connections and coverage gaps vastly complicate the graph correction procedure, and erroneous paths may remain present in the final corrected graph.

### Aligning reads to a de Bruijn graph

To align the reads to the graph a seed-and-extend approach is applied. By properly indexing the graph the seeds can be found in *O*(*m*) time, where *m* is the size of the read that is being mapped.

#### Finding maximal exact matches

To rapidly find MEMs between the nodes of the graph and the long reads, essaMEM [23] is used. These MEMs will be used as seeds for the alignment. By concatenating the sequences of every node and their reverse complement, a single sequence is constructed. From this sequence, an enhanced sparse suffix array is built by essaMEM. The sparseness factor of the index sharply reduces the space requirement for the index, compared to traditional suffix trees or enhanced suffix arrays, but this comes at the cost of a small increase in runtime.

#### Chaining seeds

To chain the seeds, several passes over the read are performed. In each iteration the algorithm considers every region of the read that has not yet been aligned. For every such region separately, the largest seeds are considered. From these seeds it is determined to which nodes the current region of the read could map. For each such node the list of all seeds between this node and the current region of the read is considered, and an optimal placement of these seeds is decided, removing the ones that do not fit. Seeds are compatible if the distance between the two seeds on the read is contained in an interval determined by the estimated error rates and the distance of the seeds in the node.

**Table 7:** Throughput and N50 for proovread on *A. hydrophila* without preprocessing the short reads with Karect

proovread without Karect	94.59 %	7303 bp
proovread with Karect	95.40 %	9803 bp

Generally, larger MEMs are less likely to be noise than shorter seeds, since the number of all *k*-mers increases exponentially if *k* increases and the number of *k*-mers contained in a sequence is similar to the size of the sequence, independent of *k*. There can still be noisy long seeds, especially when the genome contains imperfect repeats. In this case, the correct seeds can usually be recognized amidst the noisy seeds by considering the context. Firstly, the local context is considered, by comparing the seeds in the same node. This way seeds that occur in the same order in a node and in the read can be chained together to form inexact matches. Secondly, if the situation is still ambiguous, the global context is considered, by comparing the alignments in the neighborhood of the ambiguous region. If this neighborhood has not yet been chained in previous passes, the chaining of the current region is delayed to the next pass.

After obtaining the presumed layout of the seeds, the quality of the alignment is assessed. The following cases are filtered:

1. Local mappings that are not super maximal, i.e., local mappings that are on the read contained in a larger local mapping.
2. Local mappings that cover less than a predetermined fraction of the node. The absence of any seeds in the rest of the node makes it less likely that this is actually a correct mapping. The fraction can be calculated based on the work in Section .

After the local alignments are computed for the current pass, the next phase begins: chaining the alignments between different nodes by following unique paths in the graph. During this phase every local alignment is extended by considering the possible paths in the graphs. Both directions of the alignments are extended in the same manner, as follows:

1. If there is a unique edge, this edge must be correct and the local alignment is extended along this edge.
2. If there are several edges, the lengths of the end nodes are considered. Since the extension takes place between two regions of the read, certain estimates can be made for the maximal distance between the alignments, edges that are too long are then not considered.
3. If at any point there are no suitable edges to extend along, a mistake was made at some point. Either the graph is incorrect or the original local chaining was erroneous. In either case the erroneous region is reprocessed in a new local chaining step.

In the rest of this section the distance between corrected regions on a read is denoted as *n* and the estimated insertion and deletion rates of the data are denoted as *i* and *d*.

After the unique-extension step, the resulting chains may overlap in the graph, in which case they can be linked together to make one consecutive path. Overlapping chains are however not a sufficient condition for linking, the size of the sequences represented by the path and the read need to be compared. If the sequence on the path is smaller than $(1+2i)^{-1}n$, the shortest cycle at the common point is considered. If this shortest cycle can not adequately fill the gap, then the paths are not joined and the gap is left for the next pass. Likewise, if the resulting chains do not meet, the shortest path between both end points is considered. If this shortest path can not adequately fill the gap, the gap is again left for the next iteration.

By clustering seeds and only using shortest path algorithms to chain the nodes, computationally expensive path searching and per base alignment can be avoided.

#### Final alignment

After all passes of the algorithm have been performed, there are often several remaining possible alignments. The alignment that best covers the read is selected, and used for the error correction. To correct the read ends, the alignment is extended along unique paths in the graph. If the read is estimated to continue further than the longest unique path, this part of the read will be discarded. Correcting these read ends is typically an expensive operation, since they have to be aligned to all possible paths leaving the aligned path. This is the case because these read ends do not contain reliable seeds. If they would, those seeds would have been chained with the path. This could be further improved upon by searching for smaller seeds in the read ends, however, this is not done in Jabba, since it is a relatively expensive operation for a small gain.

If any of the previously discarded read ends contains an alignment, this alignment is additionally used to correct that read end. This methodology is applied recursively. In this way one read in the input can result in several smaller non-overlapping reads in the output. This allows Jabba to deal with coverage gaps in the graph, where no uninterrupted path exists. Additionally it allows Jabba to handle chimeric third generation reads.

### Settings

Jabba takes several parameters that can affect the results. Most importantly the minimal length *l* of MEMs for the initial search can be specified, the standard value is $l=20$, but this should be chosen based on the discussion in section in function of the data. If for a particular read, an extremely high or low amount of seeds are found, the seed finding procedure is repeated for this read, with a more suitable choice of *l*. Incorrectly setting this parameter may hence still lead to results comparable to a correct choice of *l*, but at the cost of an increase in runtime.

Another crucial parameter is the *k*-value of the de Bruijn graph. If *k* is too large, the graph will have many small disconnected nodes. Since Jabba only corrects to paths that actually exist in the graph, these nodes will typically not contribute anything to the error correction, and most of the second generation data is not used. If, on the other hand, *k* is too small, many small repeats remain in the graph, severely reducing the size of linear paths in the graph and increasing the path-finding complexity. Building a de Bruijn graph from corrected second generation data is a relatively inexpensive operation. As such, this parameter can be optimized by constructing several graphs with different *k*-mer sizes, and comparing the connectedness of the resulting graphs. If two graphs have a similar degree of connectedness, i.e., they contain a similar number of bases in their largest components, then the graph with the largest *k* should be preferred.

The maximal number *p* of iterations of the algorithm can be specified, the standard value is $p=5$. Finally, Jabba has two different output modes, *short* attempts to correct the read completely by estimating how far from the extremal aligned seed the alignment still continues, while *long* extends the correction maximally along linear paths in the graph. Because of this, the *long* output mode has a small risk of creating additional chimeric reads, but the resulting reads will in many cases be several times longer than the original reads. The *short* output mode results in output that is more similar to the input reads.

## Expected maximal exact matches in sequences

In this section the occurrence of maximal exact matches in reads is investigated. Insertions and deletions have a different effect on the size of maximal exact matches than substitutions. A substitution error puts a firm stop to any running exact matches, while an insertion or deletion may allow for the exact match to continue, effectively looking like an error at a further point in the read. In the following, this difference is ignored and all errors are treated like they were substitutions. Because of this, the size of MEMs is slightly underestimated for sequences that contain insertions or deletions. It is also assumed that errors are uniformly distributed in the sequences, as is the case for Pacific Biosciences SMRT reads.

### Coverage by exact regions

In this section the expected fraction of a long read that should be covered by MEMs larger than a given size is explored, under the assumption that the reference contains no errors. Variations on this topic have been explored in [24–26]. In the following, *n* is the length of the read, *p* is the error-rate and *m* the threshold for maximal exact matches. An *exact region of size k* on a read is defined as *k* correct consecutive bases in that read. The *coverage by exact regions* is the fraction of bases that are contained in exact regions.

The expected number of exact regions (including those of length 0) is the expected number of errors, i.e., *np*. The expected coverage of a read by exact regions of size *k* is then the product of (i) the coverage of the read by one exact region of size *k*: *k*/*n*, (ii) the expected number of exact regions: *np*, and (iii) the probability that an exact region has size *k*: $(1-p)^kp$. This results in:

1 $$\begin{aligned} k(1-p)^kp^2 \; . \end{aligned}$$

**Fig. 2:**
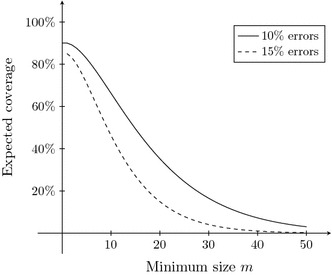
Expected coverage by exact regions of size $k\ge m$ for reads of size 10,000 with $10\,\%$ and $15\,\%$ errors, expressed as percentages of the whole read as a function of the minimal size of the exact regions

Summing (1) over all $k \ge m$ gives the expected coverage of the read by exact regions of size $k \ge m$:

2 $$\begin{aligned} \sum \limits _{k=m}^{\infty }k(1-p)^kp^2 = (1-p)-\sum \limits _{k=0}^{m-1}k(1-p)^kp^2 \; , \end{aligned}$$

the right hand side provides a finite formula to compute this expected coverage. Figure 2 shows the expected coverage by exact regions larger than *m*, for error-rates $p=10\,\%$ and $p=15\,\%$. The maximum $1-p$ is obtained at $\{0,1\}$ since every correct base is contained in an exact region of size $\ge 1$. It can be seen that increasing *p* leads to a steeper descent near the inflection point. While it was a priori clear that a lower error rate leads to larger exact regions, this also shows that the equilibrium between a sufficient amount of seeds and a sufficiently large minimal seed length, is less stable for higher error rates.

### Occurrence of exact regions

The expected length of the longest exact region in a read of size *n* is denoted by $ER_p(n)$. If $np(1-p)^m \ge 1$ then at least one exact region of size $k\ge m$ is expected in a read of size *n*, hence the expected length of the longest run can be approximated by solving $np(1-p)^m = 1$ for *m*:

3 $$\begin{aligned} ER_p(n) \approx -\log _{1-p}{np}. \end{aligned}$$

The distribution around this average can be approximated by the complement of a Gumbel distribution with cumulative distribution function

4 $$\begin{aligned} F(x) = \exp {\left( -(1-p)^{x+1}\right) }; \end{aligned}$$

the probability that a read of length *n* will have an exact region of size $k\ge m$ is then approximated by

5 $$\begin{aligned} \begin{array}{ll} P(n,p,m) &{}= 1-F(m + ER_p(n)) \\ &{}= 1-\exp {\left( -np(1-p)^{m+1}\right) }. \end{array} \end{aligned}$$

**Fig. 3:**
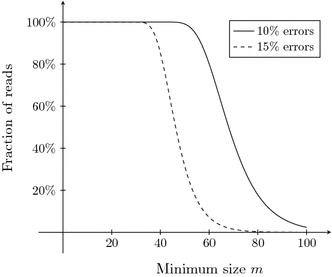
Expected percentage of reads of size 10,000 that contain at least one exact region of size $k\ge m$, for reads with $10\,\%$ and $15\,\%$ errors

These approximations are highly accurate when *p* and *n* are sufficiently large. Figure 3 shows the fraction of reads of length *n* that are expected to have an exact region of size *m*, for error-rates $p=10\,\%$ and $p=15\,\%$. For sufficiently large values of *n*, replacing *n* by $n^\prime > n$ shifts the graph to the right by a term $\log _{1-p}{{n}/{n^\prime }}$, replacing *p* by $p^\prime < p$ shifts the graph to the left and steepens the descent near the inflection point. This again shows that larger error rates make the determination of a proper seed size threshold less stable.

### Applications

During the local chaining step from section  one can apply the results of section  to decide whether a local mapping is plausible or not. For each mapping the coverage by exact regions can easily be computed by counting seed sizes. The resulting number can then be compared to the expected coverage that can be obtained from section. If there is a significant deviation in either direction, the local mapping gets a lower rating.

When computing mappings it is required to have at least one seed available, hence the results from section  propose good upper bounds for the minimum length of seeds, depending on the read size and error rates. To a certain extent this result can also be used to estimate the probability of a read containing several exact regions of a minimal size. If a read of size *n* contains a MEM of size $k \ge m$, then this MEM divides the read in two pieces, one of size $n^\prime$ and the other of approximately size $n-n^\prime$. This approximation of the piece-sizes is made since typically *k* is significantly smaller than *n*, and *k* is not known a priori. The conditional probability of the read containing a second MEM of size larger than *m* then becomes $1 - (1 - P(n^\prime , p, m))(1 - P(n - n^\prime , p, m))$, with *P* as in (5). Since $n^\prime$ depends on the read, it is a priori not known and integrating over $n^\prime$ is required. The distribution of the size of $n^\prime$ can be approximated by the uniform distribution on $\{0,\dots ,n\}$, and because of symmetry this leads to the following estimate of the a priori probability of a read of size *n* containing at least 2 exact regions with size larger than *m*:

6 $$\begin{aligned} P(n, p, m) = \frac{2}{n}\sum \limits _{n^\prime =0}^{n/2} Q(n, n^\prime , p, m)\;, \end{aligned}$$

where $Q(n, n^\prime , p, m) = 1-\big (1-P(n^\prime , p, m)\big )\big (1-P(n-n^\prime , p, m)\big )$. In a similar fashion, equation (6) can be extended to multiple seeds, possibly of different minimal sizes. However one should be careful when using (6) and other extensions of (5), since the approximation made by *P*(*n*, *p*, *m*) becomes less accurate when *n* decreases.

## Results

Jabba is compared with LoRDEC [13] and proovread [12]. In [12, 13] it is demonstrated that LoRDEC and proovread perform better than both LSC [10] and PacBioToCA [11].

### Data

To evaluate Jabba a combination of simulated and real data was used. The sources of the data are specified in Table 1.

For *Escherichia coli*, *Aeromonas hydrophila*, *Saccharomyces cerevisiae*, *Arabidopsis thaliana*, and *Drosophila melanogaster*, Illumina paired-end reads were simulated using ART Illumina [27], using the MiSeq profile. For *Ostreococcus tauri*, real Illumina reads were used, with an average size of 76 bp.

From the *A. hydrophila* genome Pacific Biosciences reads were simulated using pbsim [28], with average read length of 10 kbp and $15\,\%$ errors, distributed as $60\,\%$ insertions, $30\,\%$ deletions and $10\,\%$ substitutions. Real Pacific Biosciences datasets were used for all other genomes. For *O. tauri*, the Illumina and Pacific Biosciences data were sequenced from the same strain.

### Parameters

#### LoRDEC

LoRDEC was run with $k=19$ for the bacterial data sets, for *S. cerevisiae*, and for *O. tauri*, as suggested in [13]. For the larger genomes the best results were obtained for $k=21$. LoRDEC results are shown with and without post-processing with LoRDEC-trim. For all data sets the short reads were preprocessed with Karect, to allow a more clear comparison of the tools. Additionally, for *E. coli*, *A. hydrophila* and *O. tauri*, LoRDEC was applied to the uncorrected reads.

#### proovread

For proovread the standard parameters were used.

#### Jabba

For Jabba the minimum MEM size was $l=20$ and the de Bruijn graphs were built with $k=75$ for all datasets except for *O. tauri*, where $k=55$ was used due to the short read lengths of the second generation data, i.e., 76 bp. Jabba was run with the *short* output mode.

### Evaluation metrics

**Fig. 4:**
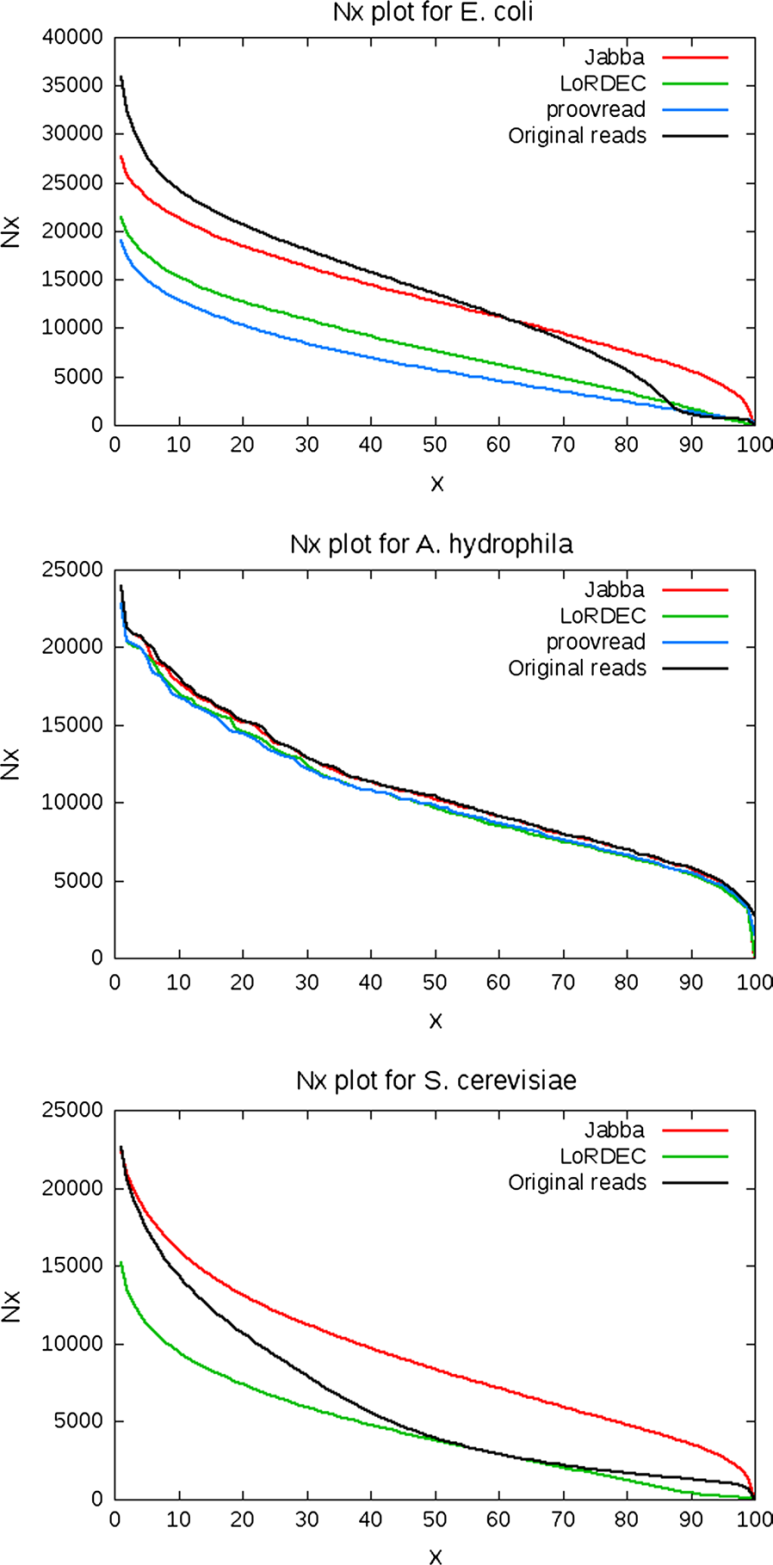
N*x* plots for *E. coli*, *A. hydrophila* and *S. cerevisiae*

**Fig. 5:**
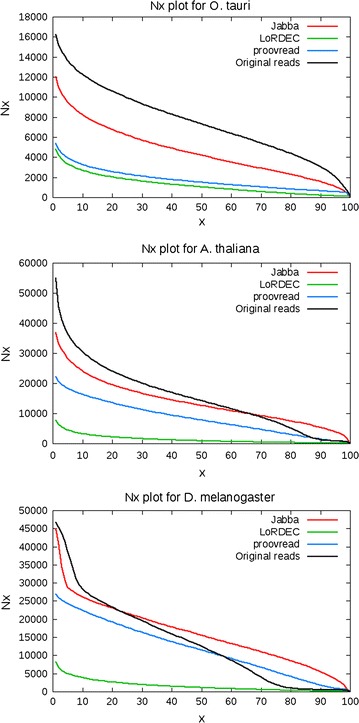
N*x* plots for *O. tauri*, *A. thaliana* and *D. melanogaster*

After correction the reads are aligned to the reference genome with BLASR [29], with a minimum alignment identity of $70\,\%$. In Table 3 the following metrics are used to compare the performance of the tools:

- Gain: relative change in errors of the aligned reads compared to the original reads.
- Accuracy: the identity percentage of the aligned reads.
- Error-free: the fraction of the aligned reads that aligns without errors.
- Aligned: the fraction of aligned bases.
- Throughput: the ratio of corrected base pairs and input base pairs.
- N*x*: the N*x* of the reads, i.e., the minimum read size such that all reads larger than this contain $x\%$ of the bases in the data set. In Table 3 the N50 is shown, continuous plots of N*x* values are displayed in Figures 4 and 5.
- CPU time: the average CPU time per read.
- Memory: the peak memory usage.

All experiments were run on dual-socket octa-core Intel Xeon Sandy Bridge computing nodes at 2.6 GHz and 64 GB of memory. The runtimes and memory usage are measured using the standard Linux time command.

### Evaluation and discussion

Table 2 shows the results for LoRDEC, proovread and Jabba. The output of LoRDEC has been post-processed by trimming and splitting the reads and only retaining the regions of the reads that are of high quality. The proovread run on *S. cerevisiae* did not finish after 3 days and is not included in this discussion. In the following discussion every reference to LoRDEC concerns the results of LoRDEC with preprocessing by Karect, unless otherwise mentioned.

On the simulated long reads for *A. hydrophila*, all tools perform very well. The main difference between the tools on the simulated data is in the percentage of error-free reads; almost all reads produced by Jabba and proovread contain no errors. LoRDEC on the other hand, only reaches up to $86.74\,\%$ error-free reads.

On all real data sets, all the tools perform worse than on the simulated data set. From the table it is clear that LoRDEC and proovread have a slightly higher throughput than Jabba on all data sets. However, a significant percentage ($11\,\%$–$50\,\%$) of the reads corrected by LoRDEC do not align to the reference. For the aligned reads, all the tools achieve over $98\,\%$ accuracy on all datasets. Jabba consistently has the highest accuracy on real data sets and keeps performing well even on the larger genomes. Both LoRDEC and proovread obtain significantly worse accuracies on the larger genomes than on the bacterial genomes. For all data sets, except for *O. tauri*, over $95\,\%$ of the Jabba-corrected reads that align to the reference contain no errors. For LoRDEC and proovread this number is significantly lower, many reads still contain errors.

In general, the output of Jabba is very reliable for both the real and the simulated data. Almost all reads that are corrected by Jabba are of very high quality, and many of them contain no errors at all.

From Figs. 4 and 5 it is clear that on every data set, the output from Jabba is contained in longer reads than the output from both other tools.

The memory usage of all tools is shown in Table 4. The memory usage of Jabba is almost linear in the genome size. LoRDEC uses more memory than Jabba on the smaller genomes, but this is a peak during the construction of the de Bruijn graph. On the two larger genomes, Jabba uses more memory than LoRDEC. The memory usage of Jabba is dominated by the storage of the enhanced sparse suffix array, which can be linearly decreased by increasing the sparseness factor. This is shown for *A. hydrophila* in Table 5. In this table the relation between memory usage (*m*) and sparseness factor (*s*) is approximately $m = {82.57}/{s} + 20.50$. This sparseness factor allows Jabba to also run on lower memory machines, but this comes at a cost in runtime. Another major contributor to the peak memory usage are reads that have an overabundance of MEMs with the graph.

The average CPU time per read is displayed in Table 3. Jabba processes 10–100 reads per CPU second. Both LoRDEC and proovread require significantly more CPU time. The high speed of Jabba is a result of the pseudo alignment approach.

The preprocessing with Karect requires a high amount of computing resources, as shown in Table 6. However, the increase in error-free reads is significant, and on all data sets LoRDEC performs better in this regard after preprocessing the second generation data with Karect. Additionally, Table 7 indicates that the throughput and N50 of proovread corrected reads can also be significantly improved by preprocessing the short reads. This indicates that the integrated short read error correction (*k*-mer frequency filtering) performs worse than the dedicated second generation error correction tool Karect. For Jabba this preprocessing step carries the additional advantage of allowing a larger *k*-mer size for the de Bruijn graph. A de Bruijn graph that was built from uncorrected short reads, with $k=75$, is very disconnected and can not be used for alignment of long reads. From the results on a perfect graph for *D. melanogaster*, it is clear that after preprocessing short reads with Karect, Jabba performs equally well on a graph built from short reads as on a perfect graph. Any further improvements to the hybrid error correction procedure should therefore be focused on the alignment procedures, and not on further correction of the second generation data.

## Conclusion

Jabba produces highly reliable corrected reads: almost all corrected reads align to the reference, and these alignments have a very high identity. Many of the aligned reads are error-free and the N50 of the reads is high compared to other tools. Additionally, Jabba corrects reads using a very low amount of CPU time. From this we conclude that pseudo alignment with MEMs is a fast and reliable method to map long highly erroneous sequences on a de Bruijn graph.

From the comparison of LoRDEC and proovread with and without preprocessing with Karect, we conclude that dedicated second generation error correction tools can provide a meaningful contribution to the hybrid error correction procedure. Especially for the creation of error-free reads, LoRDEC’s built-in short read error correction procedure performs significantly worse than building a graph from corrected short reads. Additionally, this preprocessing is vital for Jabba, since it allows Jabba to use a de Bruijn graph with a high value of *k*.

Jabba performs equally well on a perfect graph and a graph constructed from corrected short reads, future work in hybrid error correction should be focused on improving the alignment procedures.
